# Serum Redox Biomarkers in Canine Mast Cell Tumors: Exploratory Associations with Proliferative Activity and Clinicopathological Features

**DOI:** 10.3390/antiox15091142

**Published:** 2026-09-09

**Authors:** Argyrios Ginoudis, Dimitra Pardali, Mathios E. Mylonakis, Serafeim Chaintoutis, Dimitris Galiatsatos, Angelos-Lauris Thomas, Zoe Polizopoulou

**Affiliations:** 1 Diagnostic Laboratory, School of Veterinary Medicine, Faculty of Health Sciences, Aristotle University of Thessaloniki, 54627 Thessaloniki, Greece; agkinou@vet.auth.gr (A.G.); dpardali@vet.auth.gr (D.P.); schainto@vet.auth.gr (S.C.); 2 Companion Animal Clinic, School of Veterinary Medicine, Faculty of Health Sciences, Aristotle University of Thessaloniki, 54627 Thessaloniki, Greece; mmylonak@vet.auth.gr (M.E.M.); plato@vet.auth.gr (A.-L.T.); 3 School of Science and Technology, Hellenic Open University, 26221 Patras, Greece; dgaliatsatos2010@hotmail.com

**Keywords:** dog, mast cell tumor, oxidative stress, malondialdehyde, Ki-67, lymph node metastasis

## Abstract

Canine mast cell tumors (MCTs) exhibit heterogeneous biological behavior and provide a clinically relevant setting in which relationships between tumor phenotype and systemic redox homeostasis can be investigated. This exploratory prospective cohort study evaluated complementary circulating redox-related biomarkers representing hydroperoxide-derived oxidant products, antioxidant capacity, lipid peroxidation-related products, and oxidative DNA damage, and examined their associations with clinicopathological features of canine MCTs. Sixty-seven dogs with cytologically and histologically confirmed cutaneous or subcutaneous MCTs were included. Clinical staging was performed using cytological evaluation of lymph nodes, liver and spleen, and excised tumors were classified according to the Patnaik and Kiupel systems. Proliferative activity was assessed using Ki-67, and surgically excised lymph nodes were classified according to the Weishaar HN system. Serum reactive oxygen metabolites (d-ROMs), malondialdehyde (MDA), 8-hydroxy-2′-deoxyguanosine (8-OHdG) and antioxidant capacity using the OXY-adsorbent test were measured before therapeutic intervention. In the unadjusted analysis, serum MDA concentrations were higher in dogs with Ki-67 >23 than in dogs with Ki-67 ≤23 (median 10.8 vs. 7.0 μmol/L; raw *p* = 0.0254); however, this comparison did not remain statistically significant after Bonferroni correction for the 12 formal inferential biomarker × clinicopathological comparisons (adjusted *p* = 0.3052). The estimated Hodges–Lehmann location shift was +3.00 μmol/L (unadjusted bootstrap 95% CI, 0.58–6.00). No evaluated biomarker–clinicopathological association remained statistically significant after multiplicity adjustment. The observed MDA–Ki-67 pattern should therefore be regarded as exploratory and hypothesis-generating rather than as evidence of established prognostic or clinical utility. Long-term data on recurrence, progression, and survival were not available; therefore, prognostic utility could not be evaluated. Larger prospective studies incorporating longitudinal outcomes are warranted.

## 1. Introduction

Mast cell tumors (MCTs) are among the most frequently diagnosed skin neoplasms in dogs, accounting for approximately 16–21% of canine cutaneous tumors and representing a common diagnostic and therapeutic challenge in small animal practice [1,2,3]. Their biological behavior is heterogeneous: some tumors are cured by excision, whereas others recur, metastasize to regional lymph nodes and/or visceral organs, and are associated with short survival time despite multimodal therapy [2,4,5]. This heterogeneity has driven extensive investigation of histological, clinical, immunohistochemical and molecular prognostic indicators [6,7,8].

Histopathological grading remains central to prognostication. The Patnaik three-tier system has been used historically, whereas the Kiupel two-tier system was proposed to improve reproducibility and more clearly separate biologically low-risk from high-risk cutaneous MCTs [4,9]. Other established or proposed prognostic variables include mitotic index, Ki-67 and AgNOR-based proliferation indices, KIT immunohistochemical pattern, c-kit mutation status, surgical margin status, anatomic location, recurrence, and evidence of nodal or distant metastasis [6,7,8,10,11,12,13,14]. Subcutaneous MCTs are often associated with a more favorable clinical course than high-grade cutaneous MCTs, although a subset can behave aggressively and require careful evaluation of proliferation and KIT-related features [3,15,16].

Accurate staging is also important because canine MCTs may metastasize to regional lymph nodes and, in advanced disease, to the spleen, liver and bone marrow [2]. Fine-needle aspiration of regional lymph nodes and abdominal imaging-guided liver and spleen cytology are commonly used in clinical staging, particularly for tumors considered at increased metastatic risk [2,17]. Histopathological nodal assessment using the HN classification provides a structured evaluation of lymph node mast cell infiltration and has been associated with clinical outcome [18,19].

Oxidative stress is defined as a disruption of redox homeostasis in favor of oxidants, leading to the accumulation of reactive species or oxidative by-products that can damage lipids, proteins and nucleic acids [20,21,22]. In cancer biology, reactive oxygen species have a dual role. At controlled concentrations, they participate in signaling, proliferation and adaptation; at excessive concentrations, they may contribute to genomic instability, inflammatory amplification, angiogenesis, invasion and metastatic potential [23,24,25]. The relationship between oxidative stress and neoplasia is therefore complex, biomarker-dependent and context-dependent [21,22]. Redox homeostasis is a regulated process involving the generation and removal of reactive species, antioxidant defense systems, and reversible redox-dependent signaling. Reactive oxygen species are therefore not exclusively damaging molecules; at controlled concentrations, particularly hydrogen peroxide, they can modify redox-sensitive proteins and regulate cellular signaling, proliferation, metabolism, and adaptation. Conversely, excessive or inadequately controlled oxidant production can result in irreversible oxidative modification of lipids, proteins, and nucleic acids. Redox signaling, antioxidant responses, and oxidative molecular damage should therefore be considered related but biologically distinct processes [26,27].

Several oxidative stress biomarkers can be evaluated in serum. Reactive oxygen metabolites (d-ROMs) provide an estimate of hydroperoxide-derived oxidant products and have been analytically evaluated in canine samples [28,29]. Malondialdehyde (MDA) is a widely used marker of lipid peroxidation, whereas 8-hydroxy-2′-deoxyguanosine (8-OHdG) is an extensively investigated marker of oxidative DNA damage [24,30,31]. Antioxidant status can be approached through individual antioxidant enzymes or global antioxidant capacity tests, including the OXY-adsorbent assay, BAP, CUPRAC, FRAP and TAS/ABTS assays [22,32,33]. Redox-sensitive pathways involving, among others, NRF2-regulated antioxidant responses, NF-κB, MAPK, and PI3K/AKT signaling can influence cancer-cell proliferation, inflammatory signaling, and adaptation; these pathways were not directly investigated in the present study and are considered only as mechanistic context [34,35].

In canine medicine, altered circulating redox biomarkers have been described in inflammatory, infectious, renal, cardiovascular and oncologic diseases [22,36,37,38,39]. In canine oncology, oxidative stress alterations have been reported in lymphoma, mammary tumors and MCTs, although results differ among studies depending on tumor type, sample type, treatment status and biomarker selection [40,41,42,43,44,45,46]. Previous studies in dogs with MCTs suggested altered oxidant-antioxidant balance, including increased d-ROMs or altered antioxidant capacity, and our previous work identified an association between histological grade and PON-1 activity [44,45,46]. However, the association of additional lipid peroxidation and DNA oxidation biomarkers with Kiupel grade, Ki-67 proliferative activity and histopathological lymph node status remains incompletely defined. The objective of this exploratory prospective study was therefore to characterize complementary circulating redox-related biomarkers in dogs with MCTs and to investigate whether these measurements differed according to selected clinicopathological features, including tumor location, histological grade, proliferative activity, and histopathological lymph-node classification.

## 2. Materials and Methods

### 2.1. Study Population

Client-owned adult dogs with cytologically and histologically confirmed cutaneous or subcutaneous MCTs were prospectively included. Dogs were enrolled before surgery or chemotherapy. Dogs with concurrent disorders considered likely to substantially influence systemic redox biomarkers were excluded. Screening was based on medical history, physical examination including thoracic auscultation, complete blood count, serum biochemistry, and diagnostic imaging where clinically indicated. Eight dogs were excluded because of concurrent disease: two with cardiac disease, four with concurrent neoplasms (one squamous cell carcinoma, one pheochromocytoma, one mammary tumor, and one oral melanoma), and two with endocrine disorders (one with diabetes mellitus and one with hypercortisolism). Medication and supplement history were reviewed. Dogs receiving corticosteroids, NSAIDs, antioxidant supplements, or any chemotherapeutic agent within 6 months were excluded. Informed owner written consent was obtained before inclusion in the study.

### 2.2. Clinical Staging and Sample Collection

A complete history and physical examination were performed in all dogs, with particular attention to the number, size and anatomical distribution of cutaneous or subcutaneous masses and to the presence of peripheral lymphadenomegaly or clinical signs suggestive of systemic disease. Fine-needle aspiration cytology was performed on the primary tumor or tumors and on all palpable lymph nodes. Lymph nodes with cytological findings suspicious for metastatic mast cell involvement were selected for surgical excision and subsequent histopathological evaluation. Sentinel lymph-node mapping was not performed. Abdominal ultrasonography and ultrasound-guided fine-needle aspiration of the liver and spleen were performed for staging when clinically appropriate. Blood was collected from the jugular vein before surgical manipulation or therapeutic intervention. Blood was allowed to clot for approximately 30 min and was centrifuged at 3000 rpm for 10 min using a ROTOFIX 32 A centrifuge (Andreas Hettich GmbH, Tuttlingen, Germany). Serum was aliquoted and stored at −80 °C until analysis. The median storage duration before biomarker analysis was 5 months (IQR, 3–8 months; range, 1–12 months). Samples did not undergo freeze–thaw cycles; a different aliquot was selected for each analysis. Analyses were performed in a single batch for each analyte and every analyte was measured in duplicate for each sample. Samples showing visible hemolysis/lipemia were excluded.

### 2.3. Histopathology and Immunohistochemistry

Surgically excised tumors and cytologically suspicious lymph nodes were submitted for histopathological confirmation and classification as cutaneous or subcutaneous MCTs in a commercial laboratory (Vet Med Labor GmbH—Division of IDEXX Laboratories, Leipzig, Germany). Cutaneous tumors were graded according to the Patnaik and Kiupel histological grading systems. Ki-67 immunohistochemistry was performed in the same laboratory, and tumors were categorized using a cut-off of 23 positive nuclei per cm^2^ grid area [7,47]. Surgically excised lymph nodes were evaluated histologically and classified according to the Weishaar HN system. For the present analysis, HN1, HN2 and HN3 cases were included in the lymph node class comparison [19].

### 2.4. Oxidative Stress Biomarkers

Serum reactive oxygen metabolites (d-ROMs) were quantified using the commercially available d-ROMs test (Diacron International, Grosseto, Italy), according to the manufacturer’s instructions and as previously described in dogs [28]. This assay estimates the concentration of hydroperoxide-derived oxidant products, which are generated during oxidative processes. In the presence of iron ions, hydroperoxides react with the chromogenic substrate N,N-diethyl-p-phenylenediamine, producing a colored radical cation. Absorbance was measured spectrophotometrically at 505 nm using the Free Carpe Diem analyzer (Diacron International, Grosseto, Italy), and results were expressed in Carratelli units (CARR U), where 1 CARR U corresponds to 0.08 mg/dL hydrogen peroxide.

Serum antioxidant capacity was determined using the commercially available OXY-adsorbent test (Diacron International, Grosseto, Italy), according to the manufacturer’s instructions and as previously used in canine serum samples [39]. This assay evaluates the ability of serum antioxidants to neutralize an excess of hypochlorous acid (HClO). After incubation with HClO, the residual oxidant reacts with an alkyl-substituted aromatic amine in a chromogenic solution, producing a colored compound. The color intensity, measured spectrophotometrically with the Free Carpe Diem analyzer (Diacron International, Grosseto, Italy), is inversely proportional to the total antioxidant capacity of the sample. Results were expressed as micromoles of HClO consumed per milliliter of serum (μmol HClO/mL).

Serum malondialdehyde (MDA) concentration was measured using a commercially available colorimetric assay based on the thiobarbituric acid (TBA) method (Invitrogen Malondialdehyde (MDA) Colorimetric Assay Kit, Cat. No. EEA015, Thermo Fisher Scientific, Waltham, MA, USA; Bender MedSystems GmbH, Vienna, Austria), according to the manufacturer’s instructions. The assay has a stated detection range of 2.92–40 μmol/L and an analytical sensitivity of 1.13 μmol/L. The concentrations observed in the present cohort were within the range expected from previous studies using TBA-based MDA measurements in canine serum, including recently established reference data in healthy dogs [48]. Four samples yielded concentrations below the lower limit of the stated analytical range (2.92 μmol/L); for statistical analysis, these values were assigned a value of 2.92 μmol/L. MDA, a final product of lipid peroxidation, reacts with TBA under high-temperature acidic conditions to form a red-colored MDA–TBA adduct. After incubation and centrifugation, the absorbance of the supernatant was measured at 532 nm using a Multiskan FC microplate photometer (Thermo Fisher Scientific Oy, Vantaa, Finland). MDA concentrations were calculated from the standard curve and expressed as μmol/L.

Serum 8-hydroxy-2′-deoxyguanosine (8-OHdG) concentration was determined using a commercially available competitive enzyme-linked immunosorbent assay (ELISA) kit (Invitrogen 8-OHdG Competitive ELISA Kit, Cat. No. EEL004, Thermo Fisher Scientific; Bender MedSystems GmbH, Vienna, Austria), according to the manufacturer’s instructions. The assay uses a calibration curve spanning 0–100 ng/mL, with non-zero calibrators from 1.56 to 100 ng/mL, and has a reported analytical sensitivity of 0.94 ng/mL. Canine serum 8-OHdG concentrations within this concentration range have previously been reported using competitive ELISAs, including concentrations of approximately 55 ng/mL in dogs with transmissible venereal tumors and approximately 9–16 ng/mL in senior dogs [49,50]. 8-OHdG, an oxidized derivative of deoxyguanosine, was used as a marker of oxidative DNA damage. Standards and serum samples were added to antigen-coated microplate wells together with a biotinylated detection antibody, followed by horseradish peroxidase conjugate and substrate reagent. The reaction was stopped, and absorbance was measured at 450 nm using a Multiskan FC microplate photometer (Thermo Fisher Scientific Oy, Vantaa, Finland). Concentrations were calculated from a four-parameter standard curve and expressed as ng/mL. Samples yielding concentrations above the upper limit of the calibration curve were diluted 1:10 with the manufacturer-provided sample diluent and re-assayed, and the final concentration was corrected for the dilution factor.

All assays were performed on stored serum samples. Laboratory analyses were conducted blinded to histopathological grade, lymph node metastatic status, and Ki-67 category.

### 2.5. Statistical Analysis

Data were assessed for distribution and summarized as median and interquartile range (IQR) or as mean and standard deviation, where appropriate. Categorical variables were summarized as counts and percentages. Distributional assumptions were evaluated separately for each biomarker within each comparison group using the Shapiro–Wilk test. Variance structure was additionally assessed using the median-centered Levene (Brown–Forsythe) test. For two-group comparisons in which both groups showed no statistically significant departure from normality, Welch’s independent-samples *t*-test was used as a variance-robust parametric procedure. When at least one comparison group showed evidence of non-normality, the Mann–Whitney U test was used. Because the Patnaik grade I and HN1 categories each contained only one dog, formal inferential comparisons across Patnaik and HN categories were not considered statistically reliable; these data were therefore summarized descriptively only.

All statistical tests were two-sided. Formal inferential testing was performed for the four biomarkers across three clinicopathological comparison domains (Kiupel grade, tumor location, and Ki-67 category), yielding 12 inferential comparisons. Patnaik-grade and HN-class data were excluded from formal hypothesis testing because the Patnaik grade I and HN1 groups each contained a single observation. To control the family-wise type I error rate across the 12 inferential comparisons, Bonferroni correction was applied [51]. Unadjusted *p* values are presented for transparency together with Bonferroni-adjusted *p* values. The corresponding Bonferroni significance threshold for the unadjusted *p* values was α = 0.00417. Findings observed only in the unadjusted analyses but not retained after correction were interpreted as exploratory and hypothesis-generating rather than confirmatory.

For the MDA comparison between Ki-67 categories, the magnitude of the between-group difference was additionally quantified using the Hodges–Lehmann estimator of location shift with a bootstrap 95% confidence interval. Rank-biserial correlation was calculated as a standardized non-parametric effect-size measure. Bootstrap confidence intervals were based on 50,000 resamples stratified by Ki-67 category and were intended to quantify effect magnitude rather than to replace the multiplicity-adjusted inference.

To further explore whether the relationship between MDA and Ki-67 persisted after adjustment for available potential confounding variables, a parsimonious exploratory multivariable linear regression model with natural-log-transformed MDA as the dependent variable was fitted. The primary model included Ki-67 category (>23 versus ≤23), age, sex, and tumor location as predictors. One dog with an uninterpretable sex code was excluded from this analysis, resulting in 66 complete cases. Heteroscedasticity-consistent HC3 standard errors were used, and regression coefficients were exponentiated and reported as ratios of expected MDA concentrations with 95% confidence intervals. Histological grade was not included in the full-cohort model because Kiupel grading was not applicable to subcutaneous MCTs. As a sensitivity analysis restricted to cutaneous MCTs, continuous Ki-67 was evaluated together with age, sex, and Kiupel grade. All regression analyses were considered exploratory. Statistical reporting followed standard recommendations for transparent reporting of clinical and laboratory biomarker data [52].

### 2.6. Ethical Approval

The study was conducted in accordance with the Declaration of Helsinki, and was reviewed and approved by the Bioethics Committee of the Department of Veterinary Medicine, Aristotle University of Thessaloniki (protocol code 22/01/2024/39070 and date of approval 22 January 2024).

## 3. Results

### 3.1. Epidemiological Data of the Study Population and Mast Cell Tumors Features

Sixty-seven dogs with an equal number of MCTs were enrolled. Median age was 9.0 years (range 3–14 years). Forty-four dogs were females (65.7%), and twenty-three were males (34.3%). Thirty dogs were mixed-breed (44.8%), and 37 (55.2%) dogs were purebred (Table 1). Based on histopathology, 58 tumors were cutaneous and 9 were subcutaneous. Based on the Kiupel system, thirty-six cutaneous MCTs were low grade and twenty-two were high grade; nine subcutaneous tumors were not assigned a Kiupel grade. According to the Patnaik system, one cutaneous tumor was grade I, 41 were grade II and 16 were grade III; the nine subcutaneous tumors were not assigned a Patnaik grade. Regional or distant metastatic disease was recorded in 27 dogs, while 40 dogs had no evidence of metastasis. Histopathological lymph node evaluation was available for all 27 dogs and included one HN1, 12 HN2 and 14 HN3 lymph nodes. Twenty-two dogs had Ki-67 >23, while 45 had Ki-67 ≤23. Detailed descriptive data are shown in Table 1.

### 3.2. Overall Oxidative Biomarker Results

Median (IQR) d-ROMs concentration was 95.0 (81.0–108.5) CARR U, median OXY-adsorbent capacity was 274.3 (231.7–308.8) µmol HClO/mL, median 8-OHdG concentration was 63.7 (44.7–82.0) ng/mL, and median MDA concentration measured by the TBA-based assay was 8.9 (5.4–12.2) µmol/L. Descriptive biomarker values by clinicopathological variable and, where formal inferential testing was performed, the corresponding unadjusted and Bonferroni-adjusted *p* values are summarized in Table 2.

Across the 12 formal inferential biomarker × clinicopathological comparisons, the smallest unadjusted *p* value was observed for serum MDA measured by the TBA-based assay according to Ki-67 category (raw *p* = 0.0254). However, this comparison did not remain statistically significant after Bonferroni correction for multiple testing (adjusted *p* = 0.3052). None of the 12 inferential comparisons remained statistically significant after multiplicity adjustment. Patnaik-grade and HN-class biomarker values were summarized descriptively because each included a singleton subgroup. The statistical test applied to each inferential comparison is reported explicitly in Table 2.

**Table 2:** Serum redox biomarker concentrations according to clinicopathological variables, with unadjusted and Bonferroni-adjusted *p* values for formal inferential comparisons.

Biomarker	Group 1	Group 2	Group 3	Statistical Test	Raw *p*	Bonferroni-Adjusted *p*
**Kiupel grade**	**Low grade (n = 36)**	**High grade (n = 22)**				
d-ROMs (CARR U)	92.5 (81.5–106.2)	100.5 (81.5–108.8)		Welch *t*-test	0.3871	1.0000
OXY-adsorbent	269.2 (237.8–312.0)	274.8 (213.0–300.6)		Mann–Whitney U	0.4562	1.0000
8-OHdG	64.1 (47.0–76.4)	63.5 (42.9–122.6)		Mann–Whitney U	0.5694	1.0000
MDA measured by the TBA-based assay	7.2 (5.3–10.8)	10.9 (7.0–15.8)		Mann–Whitney U	0.0512	0.6144
**Patnaik grade**	**Grade I (n = 1)**	**Grade II (n = 41)**	**Grade III (n = 16)**			
d-ROMs	105.0	91.0 (80.0–106.0)	101.5 (98.8–110.5)	Descriptive only		
OXY-adsorbent	289.1	274.3 (238.9–310.7)	258.4 (212.5–299.2)	Descriptive only		
8-OHdG	70.2	64.4 (49.1–81.6)	50.7 (39.7–89.4)	Descriptive only		
MDA	6.8	7.8 (5.4–11.6)	9.6 (6.5–16.0)	Descriptive only		
**HN class**	**HN1 (n = 1)**	**HN2 (n = 12)**	**HN3 (n = 14)**			
d-ROMs	87.0	94.5 (81.8–103.0)	102.5 (100.0–115.8)	Descriptive only		
OXY-adsorbent	244.6	277.9 (237.2–292.7)	284.1 (215.8–306.8)	Descriptive only		
8-OHdG	52.5	52.6 (45.1–90.5)	58.5 (38.2–110.2)	Descriptive only		
MDA	6.1	8.9 (6.0–10.7)	12.2 (8.7–19.7)	Descriptive only		
**Tumor location**	**Cutaneous (n = 58)**	**Subcutaneous (n = 9)**				
d-ROMs	96.5 (81.2–108.8)	90.0 (76.0–107.0)		Welch *t*-test	0.6630	1.0000
OXY-adsorbent	271.6 (226.7–307.9)	284.7 (262.0–314.2)		Mann–Whitney U	0.4292	1.0000
8-OHdG	64.1 (44.3–81.0)	62.9 (45.5–97.6)		Mann–Whitney U	0.9927	1.0000
MDA	8.7 (5.5–12.9)	9.5 (2.9–10.5)		Mann–Whitney U	0.3714	1.0000
Ki-67 category	**≤23 (n = 45)**	**>23 (n = 22)**				
d-ROMs	92.0 (80.0–104.0)	100.5 (81.5–114.5)		Welch *t*-test	0.1626	1.0000
OXY-adsorbent	272.1 (234.7–314.2)	284.9 (215.8–300.8)		Mann–Whitney U	0.6072	1.0000
8-OHdG	63.7 (45.6–79.1)	63.5 (41.0–110.4)		Welch *t*-test	0.4581	1.0000
MDA	7.0 (5.1–10.7)	10.8 (8.3–14.4)		Mann–Whitney U	0.0254	0.3052

Note. Values are presented as median (interquartile range), unless otherwise indicated. Formal inferential analyses were restricted to Kiupel grade, tumor location, and Ki-67 category. Patnaik grade and HN class were summarized descriptively because the Patnaik grade I and HN1 categories each contained only one dog. Consequently, these comparisons were not included in the multiplicity correction. Raw *p* values are reported together with Bonferroni-adjusted *p* values for the 12 formal inferential biomarker × clinicopathological comparisons. The Bonferroni-corrected family-wise significance threshold corresponds to α = 0.00417 for the unadjusted *p* values. No inferential comparison remained statistically significant after multiplicity adjustment. HN, histopathological lymph node classification; MDA, malondialdehyde; 8-OHdG, 8-hydroxy-2′-deoxyguanosine.

### 3.3. Association of Oxidative Stress Biomarkers with Histological Grade of Cutaneous Mast Cell Tumors

**Figure 1:**
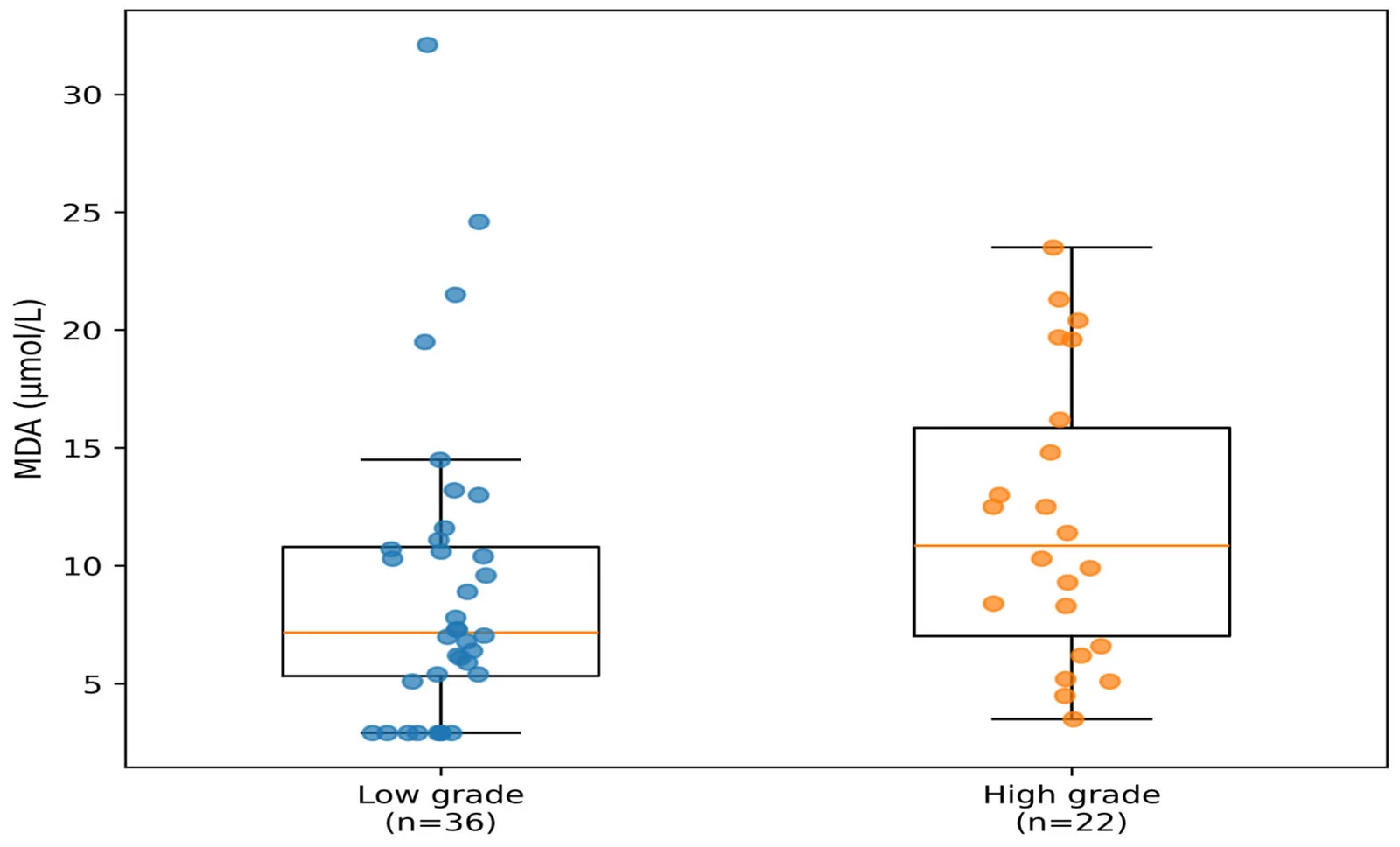
Serum MDA concentrations measured by the TBA-based assay according to Kiupel histological grade. Individual observations are superimposed on the boxplots. MDA concentrations were numerically higher in high-grade than in low-grade tumors, but the comparison was not statistically significant (raw *p* = 0.0512; Bonferroni-adjusted *p* = 0.6144).

No statistically significant differences were detected for d-ROMs, OXY-adsorbent capacity, or 8-OHdG between low- and high-grade Kiupel tumors. MDA concentrations were numerically higher in high-grade than in low-grade Kiupel tumors (median 10.9 vs. 7.2 μmol/L); however, the difference was not statistically significant (raw *p* = 0.0512; Bonferroni-adjusted *p* = 0.6144) (Figure 1).

### 3.4. Association with Lymph Node Status and Metastatic Disease

**Figure 2:**
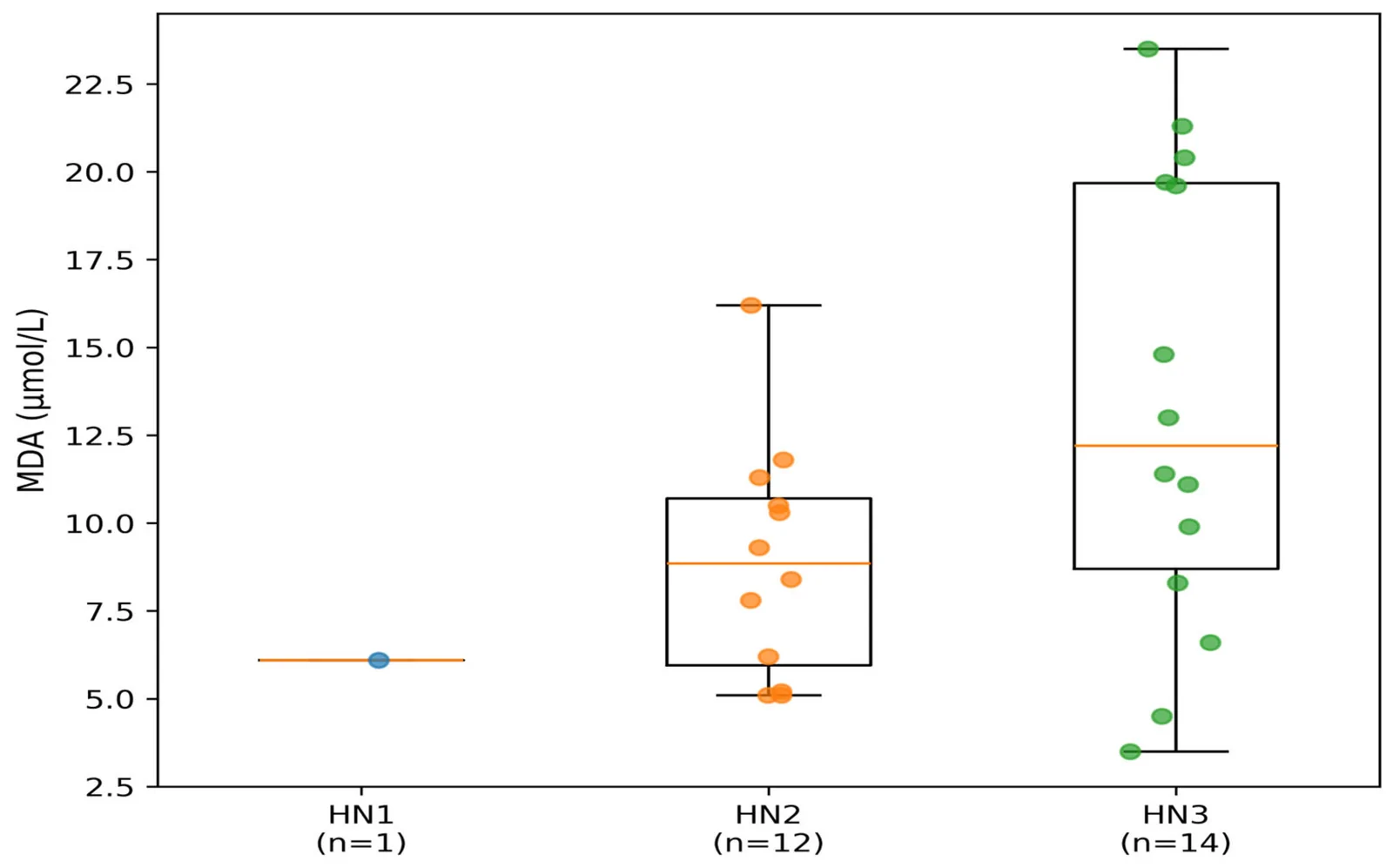
Serum MDA concentrations measured by the TBA-based assay according to histopathological lymph node classification. Individual observations are shown for HN1 (n = 1), HN2 (n = 12), and HN3 (n = 14). Median MDA concentrations were 6.1, 8.9, and 12.2 μmol/L, respectively. Because HN1 contained only one dog, no formal inferential comparison across HN categories was performed.

Biomarker concentrations according to histopathological lymph node classification are presented descriptively in Table 2. Median MDA concentrations measured by the TBA-based assay were 6.1, 8.9, and 12.2 μmol/L in HN1, HN2, and HN3, respectively. However, the HN1 category contained only one dog (HN1, n = 1; HN2, n = 12; HN3, n = 14), precluding a statistically reliable conventional comparison across the three HN categories. Accordingly, no formal inferential test was performed for HN classification, and the observed differences should be regarded as descriptive only (Figure 2).

### 3.5. Association of Oxidative Stress Biomarkers with Tumor Location and Ki-67 Category

**Figure 3:**
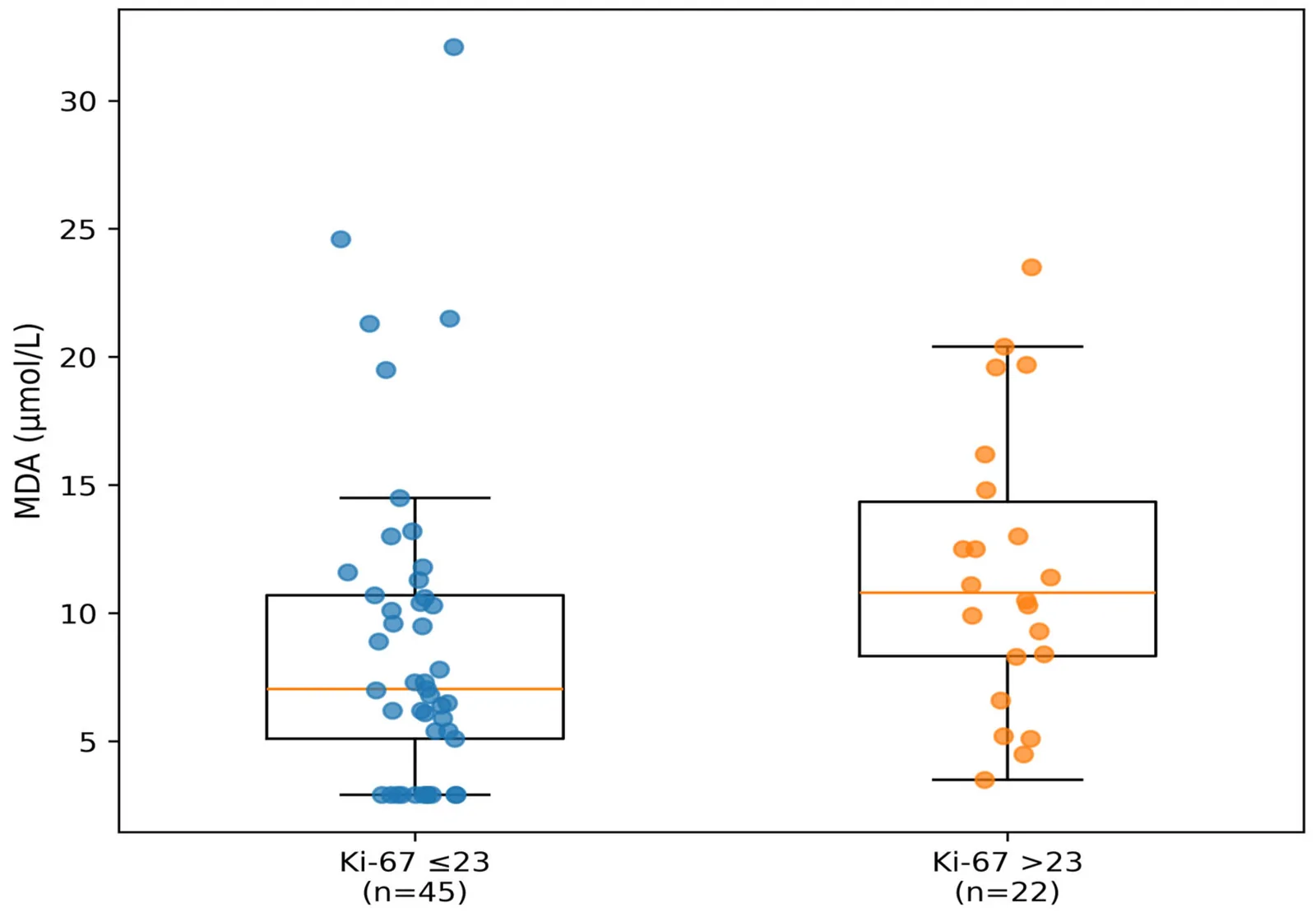
Serum MDA concentrations measured by the TBA-based assay according to Ki-67 category. Individual observations are superimposed on the boxplots. MDA concentrations were higher in dogs with Ki-67 >23 than in dogs with Ki-67 ≤23 in the unadjusted analysis (median 10.8 vs. 7.0 μmol/L; raw *p* = 0.0254), but this comparison was not statistically significant after Bonferroni correction for the 12 formal inferential comparisons (adjusted *p* = 0.3052).

No statistically significant differences were detected between cutaneous and subcutaneous MCTs for the evaluated redox biomarkers. However, this comparison was exploratory and limited by the small number of subcutaneous tumors (n = 9); therefore, the absence of statistical significance should not be interpreted as evidence of equivalence between tumor locations. In the unadjusted analysis, dogs with Ki-67 >23 had higher serum MDA concentrations measured by the TBA-based assay than dogs with Ki-67 ≤23, as presented in Figure 3 (median 10.8 vs. 7.0 μmol/L; raw *p* = 0.0254). However, this comparison did not remain statistically significant after Bonferroni correction for the 12 formal inferential biomarker × clinicopathological comparisons (adjusted *p* = 0.3052). Accordingly, this finding was considered exploratory and hypothesis-generating rather than confirmatory. The estimated Hodges–Lehmann location shift in MDA measured by the TBA-based assay for dogs with Ki-67 >23 relative to those with Ki-67 ≤23 was +3.00 μmol/L (unadjusted bootstrap 95% CI, 0.58 to 6.00 μmol/L). The corresponding rank-biserial correlation was 0.338 (bootstrap 95% CI, 0.062–0.594), providing an unadjusted standardized estimate of effect magnitude. These confidence intervals describe the magnitude and uncertainty of the unadjusted effect and do not alter the nonsignificant result after multiplicity correction. No statistically significant differences between Ki-67 categories were identified for d-ROMs, OXY-adsorbent capacity, or 8-OHdG.

### 3.6. Effect-Size Estimation and Exploratory Multivariable Analysis of MDA and Ki-67

In the primary exploratory multivariable model, 66 dogs with complete data for age, sex, tumor location, Ki-67 category, and MDA measured by the TBA-based assay were included. After adjustment for age, sex, and tumor location, Ki-67 >23 was associated with an estimated 1.34-fold MDA concentration measured by the TBA-based assay relative to Ki-67 ≤23 (95% CI, 0.99–1.81; *p* = 0.060). Thus, the association between Ki-67 category and MDA was not statistically significant after adjustment for these potential confounders. Age, sex, and tumor location were also not independently associated with MDA measured by the TBA-based assay in this exploratory model. Model explanatory performance was limited (R^2^ = 0.088; adjusted R^2^ = 0.028).

In a sensitivity analysis restricted to cutaneous MCTs, continuous Ki-67 was examined together with age, sex, and Kiupel grade. Among 57 dogs with complete data, a 10-unit increase in Ki-67 was associated with an estimated MDA measured by the TBA-based assay ratio of 0.99 (95% CI, 0.90–1.08; *p* = 0.769). This analysis therefore provided no evidence of an independent association between continuous Ki-67 and serum MDA after adjustment for these variables as presented in Table 3.

**Table 3:** Effect-size and exploratory multivariable analyses of the relationship between serum MDA and Ki-67.

Analysis	n	Effect Estimate	95% CI	*p* Value
MDA measured by the TBA-based assay, Ki-67 >23 vs. ≤23: Hodges–Lehmann location shift	67	+3.00 μmol/L	0.58 to 6.00	0.0254 ^1^
Rank-biserial correlation	67	0.338	0.062 to 0.594	
Multivariable regression: Ki-67 >23 vs. ≤23 ^2^	66	MDA ratio 1.34	0.99 to 1.81	0.060
Cutaneous-only sensitivity: continuous Ki-67, per 10-unit increase ^3^	57	MDA ratio 0.99	0.90 to 1.08	0.769

Note. ^1^ Unadjusted Mann–Whitney *p* value; the corresponding Bonferroni-adjusted *p* value for the 12 formal inferential biomarker × clinicopathological comparisons was 0.3052. Effect-size confidence intervals are unadjusted bootstrap 95% confidence intervals and are presented to quantify effect magnitude. ^2^ Exploratory log-linear regression adjusted for age, sex, and tumor location; coefficients were exponentiated and are presented as ratios of expected MDA concentrations. ^3^ Cutaneous MCTs only; exploratory log-linear regression adjusted for age, sex, and Kiupel grade.

## 4. Discussion

The most prominent finding in the unadjusted analyses was a difference in serum MDA concentrations between the predefined Ki-67 categories. Dogs with Ki-67 >23 had higher median MDA concentrations than dogs with Ki-67 ≤23; however, this finding did not remain statistically significant after correction for multiple testing. None of the 12 formal inferential biomarker × clinicopathological comparisons remained statistically significant after Bonferroni adjustment. Consequently, the observed MDA–Ki-67 pattern should be interpreted as exploratory and hypothesis-generating rather than as confirmatory evidence of an association between systemic lipid peroxidation and tumor proliferative activity [22,23,24,25].

The effect-size analysis yielded an unadjusted Hodges–Lehmann location shift of approximately 3.0 μmol/L between Ki-67 categories. However, this estimate should be interpreted together with the multiplicity-adjusted analysis. Furthermore, the Ki-67 category was not statistically associated with MDA after adjustment for age, sex, and tumor location in the exploratory multivariable model (MDA ratio 1.34, 95% CI 0.99–1.81; *p* = 0.060). A cutaneous-only sensitivity model using continuous Ki-67 and additionally accounting for Kiupel grade also showed no independent association. Collectively, these analyses indicate that the observed univariable MDA–Ki-67 signal is not sufficiently robust to support an independent relationship on the basis of the present dataset.

The absence of concordant changes across the four biomarkers should not be interpreted as evidence for the absence of altered redox biology. The assays interrogate different biochemical processes: d-ROMs primarily reflect circulating hydroperoxide-derived products, OXY-adsorbent capacity reflects the ability of serum constituents to neutralize an oxidant challenge, TBA-reactive MDA-equivalent concentrations provide an indirect estimate of lipid oxidation-related products, and serum 8-OHdG reflects circulating oxidative DNA-damage-related material. These biomarkers differ in source, molecular compartment, kinetics, clearance, and analytical specificity. Consequently, a numerical signal in one marker without parallel changes in the others may indicate pathway- or compartment-specific biology rather than a generalized systemic oxidative-stress phenotype.

Ki-67 is a well-established proliferation marker in canine cutaneous MCTs and has been associated with mortality, recurrence and metastasis in multiple studies [7,8,11,13]. The higher MDA values observed in the Ki-67 >23 group provide a preliminary hypothesis that oxidative lipid damage may accompany more proliferative tumor phenotypes. However, this interpretation is weakened by the lack of statistical significance after multiplicity correction and by the absence of an independent association in the exploratory adjusted analyses. Similar links between oxidative damage and proliferative or aggressive tumor behavior have been reported in human breast, ovarian and colorectal cancers, where MDA, 8-OHdG or composite oxidative stress measures have been associated with adverse clinicopathological features or survival [31,53,54,55]. Although median MDA concentrations were numerically higher in high-grade Kiupel tumors, the difference was not statistically significant and should not be regarded as corroborative evidence of an association with histological grade. The Kiupel system was designed to identify high-risk cutaneous MCTs using histological criteria associated with aggressive behavior and improved prognostic discrimination compared with older grading approaches [9]. Because high-grade MCTs generally have higher mitotic activity, greater risk of metastasis and shorter survival, an association with lipid peroxidation is biologically plausible [4,6,8,9]. The difference between Kiupel grades was not statistically significant (raw *p* = 0.0512) and should therefore be interpreted as a descriptive, hypothesis-generating observation rather than evidence of an association with histological grade.

The numerical differences in median MDA concentrations across HN categories are descriptive only. Formal inferential testing was not undertaken because the HN1 category consisted of a single dog. These observations should therefore be considered hypothesis-generating and should not be regarded as evidence of an association with lymph node class. Regional lymph node involvement is an important component of staging and prognostication in canine MCTs [2,18]. Histopathological lymph node classification provides a more structured assessment than cytology alone and has been correlated with outcome [19]. The descriptive ordering of MDA values across HN categories may warrant investigation in larger cohorts with adequate representation of each nodal category, but no inference regarding an association with metastatic progression can be made from the present data.

In contrast to MDA, d-ROMs did not differ significantly among histological grades, HN classes, tumor locations or Ki-67 categories, consistent with previous findings [46]. The d-ROMs test estimates circulating reactive oxygen metabolites, primarily hydroperoxides, and has been used in canine plasma and in several canine diseases [22,28,29,37]. Finotello and colleagues reported increased d-ROMs in dogs with MCTs compared with healthy controls, but that design addressed disease versus health rather than within-MCT clinicopathological categories [45]. The lack of association in the present study may indicate that d-ROMs reflect generalized oxidative burden rather than specific tumor aggressiveness, or that serum hydroperoxide concentrations are affected by preanalytical and biological variability.

Serum 8-OHdG was also not associated with the evaluated clinicopathological variables. 8-OHdG is a marker of oxidative DNA damage and has been associated with cancer risk or outcome in some human malignancies [24,31,54]. However, circulating concentrations may not directly mirror oxidative DNA damage occurring within tumor tissue. Studies in canine mammary neoplasia suggest that oxidative stress findings may differ between blood and tumor tissue, emphasizing the importance of sample matrix when interpreting redox biomarkers [22,41,43]. Future paired serum and tissue-based studies may clarify whether DNA oxidation is locally relevant in canine MCTs even when serum 8-OHdG is uninformative.

OXY-adsorbent capacity was also not associated with the evaluated clinicopathological variables. This assay provides a composite functional estimate of circulating non-enzymatic antioxidant capacity by measuring the ability of serum antioxidants to neutralize a standardized hypochlorous-acid challenge, rather than quantifying individual antioxidant molecules or enzymatic antioxidant defenses [39,56]. Therefore, an unchanged OXY-adsorbent result does not exclude alterations in intracellular antioxidant regulation or in specific systems such as glutathione metabolism, superoxide dismutases, catalase, glutathione peroxidases, or NRF2-regulated antioxidant responses [57]. Cancer cells may adapt to increased oxidative stress through coordinated changes in these antioxidant and redox-regulatory pathways, and such local adaptations within tumor tissue may occur without a corresponding detectable alteration in systemic antioxidant capacity [57].

The present findings should be considered alongside those of previous canine oncology studies. Dogs with lymphoma have been reported to show altered oxidant–antioxidant profiles, and some studies have linked oxidative markers with disease stage, immunophenotype, tumor burden or treatment response [40,42,44]. Dogs with mammary tumors have also shown changes in lipid peroxidation, antioxidant enzymes, total antioxidant capacity or inflammatory biomarkers, although results vary according to tumor biology and sample type [41,43,58]. In canine MCTs, Finotello and colleagues reported increased oxidative status and reduced antioxidant capacity, Cucchi and colleagues evaluated oxidant–antioxidant profiles in primary cutaneous MCT, and our previous work suggested that PON-1 activity may be associated with histological grade [44,45,46]. The current results extend this field by focusing on MDA and 8-OHdG in a larger cohort and by including HN lymph node classification.

No statistically significant differences in circulating redox biomarkers were detected between cutaneous and subcutaneous tumors in this exploratory comparison. This was somewhat unexpected because subcutaneous MCTs are often considered to have a more favorable prognosis than many cutaneous high-grade tumors [3,15,16]. However, subcutaneous MCTs are biologically heterogeneous and the number of subcutaneous tumors in this cohort was small. In addition, circulating redox biomarkers may be insufficiently sensitive to distinguish anatomical tumor compartments unless the biological differences are accompanied by substantial systemic oxidative changes. Given the small number of subcutaneous MCTs (n = 9), the lack of statistical significance should not be interpreted as evidence of biological equivalence between cutaneous and subcutaneous tumors or their circulating redox profiles.

The present findings do not establish MDA as a preoperative prognostic or risk-stratification biomarker. Although MDA showed the strongest unadjusted association among the evaluated biomarker comparisons, this finding did not remain significant after multiplicity correction and was not independently associated with Ki-67 in the exploratory adjusted analyses. The results therefore support further investigation of MDA as a biologically relevant candidate marker rather than current clinical application. Demonstration of clinical utility would require appropriately powered prospective studies incorporating longitudinal outcomes, multivariable prognostic modeling, assessment of incremental predictive value, and independent validation [2,3,7,9,13,19].

This study has several limitations. It did not include a healthy control group, and therefore cannot determine whether dogs with MCTs had higher oxidative stress than healthy dogs. The aim was instead to evaluate associations within the MCT population. Some clinically relevant subgroups, especially high-grade tumors, subcutaneous tumors and higher HN categories, were relatively small. Only serum biomarkers were evaluated; tumor tissue concentrations of MDA, 8-OHdG and antioxidant enzymes may provide different or complementary information. The study also involved multiple biomarker × clinicopathological comparisons. Although Bonferroni correction was applied to the 12 formal inferential comparisons, isolated findings observed in the unadjusted analyses should be interpreted cautiously and regarded as exploratory and hypothesis-generating. Oxidative stress biomarkers can be influenced by inflammation, diet, age, exercise, comorbidities, sample handling and analytical method, although our study design managed to minimize those effects through the exclusion criteria and standardized sampling and processing methods [22,25,28,32]. The MDA-related findings also require consideration of the analytical limitations of the TBA method. Although widely used as an index of lipid peroxidation, the TBA reaction is not specific for endogenous MDA, because additional aldehydes and other TBA-reactive compounds may contribute to the measured chromogenic signal. The values obtained in this study should therefore be interpreted as TBA-reactive MDA-equivalent concentrations rather than as a highly specific quantification of circulating endogenous MDA. More selective approaches, including chromatographic or mass-spectrometric methods and measurement of F2-isoprostanes, would strengthen future investigations of lipid oxidative damage. Finally, the study did not include long-term follow-up for recurrence, progression-free interval or survival, which limits conclusions regarding prognostic value.

Future investigations should include larger prospective cohorts, healthy and disease controls, standardized sampling conditions, paired serum and tumor tissue measurements, and longitudinal follow-up. Multivariable models incorporating histological grade, mitotic index, Ki-67, HN class, KIT/c-kit variables and redox biomarkers would be required to determine whether MDA provides independent prognostic information beyond established clinicopathological factors [8,9,19]. Additional markers, such as protein carbonyls, AOPP, isoprostanes, glutathione-related analytes, PON-1, CUPRAC and FRAP, could also be included to better define which oxidative pathways are most relevant in canine MCTs [22,33,46,59].

## 5. Conclusions

In this exploratory prospective cohort of dogs with MCTs, none of the evaluated serum redox biomarker–clinicopathological associations remained statistically significant after correction for multiple testing. TBA-reactive MDA-equivalent concentrations were numerically higher in dogs with Ki-67 >23 in the unadjusted analysis, generating a hypothesis regarding lipid oxidation-related processes and tumor proliferative activity; however, this pattern was not supported by the multiplicity-adjusted or exploratory multivariable analyses. The findings therefore do not establish a generalized systemic oxidative-stress phenotype, independent prognostic value, or clinical utility. Larger studies incorporating analytically validated redox measurements, standardized preanalytical conditions, paired serum and tumor-tissue analyses, assessment of antioxidant and redox-regulatory pathways, and longitudinal outcomes are warranted.

## Figures and Tables

**Table 1:** Epidemiological data of 67 dogs with cutaneous or subcutaneous mast cell tumors included in the study.

Variable	Value
**Dogs included**	67 (100.0%)
**Age, years, median (range)**	9.0 (3.0–14.0)
**Sex**	
Female	44 (65.7%)
Male	23 (34.3%)
**Breed**	
Mixed-breed	30 (44.8%)
Pitbull	8 (11.9%)
Labrador Retriever	4 (6.0%)
Boxer	4 (6.0%)
Maltese	4 (6.0%)
Yorkshire Terrier	3 (4.5%)
French Bulldog	3 (4.5%)
Caucasian Shepherd	2 (3.0%)
Shar-Pei	1 (1.5%)
Cocker Spaniel	1 (1.5%)
Epagneul Breton	1 (1.5%)
Golden Retriever	1 (1.5%)
Schnauzer	1 (1.5%)
Cane Corso	1 (1.5%)
Dogo Argentino	1 (1.5%)
American Staffordshire Terrier	1 (1.5%)
English Setter	1 (1.5%)
**Tumor location**	
Cutaneous	58 (86.6%)
Subcutaneous	9 (13.4%)
**Kiupel grade**	
Low grade	36 (53.7%)
High grade	22 (32.8%)
Not applicable (subcutaneous)	9 (13.4%)
**Patnaik grade**	
Grade I	1 (1.5%)
Grade II	41 (61.2%)
Grade III	16 (23.9%)
Not applicable (subcutaneous)	9 (13.4%)
**Metastatic disease**	
Yes	27 (40.3%)
No	40 (59.7%)
**Histopathological lymph node classification**	
HN1	1 (1.5%)
HN2	12 (17.9%)
HN3	14 (20.9%)
Not applicable (non-metastatic disease)	40 (59.7%)
**Ki-67 category**	
≤23	45 (67.2%)
>23	22 (32.8%)
Ki-67 index, median (IQR)	8.8 (4.7–27.4)
Mitotic figures, median (IQR)	2.0 (1.0–6.0)

Percentages are based on the total population (*n* = 67). One sex code was not interpretable in the source spreadsheet. Kiupel and Patnaik grading were applied to cutaneous tumors; subcutaneous tumors were categorized as not applicable.

## Data Availability

The data presented in this study are available on request from the corresponding author due to ethical restrictions.

## Abbreviations

The following abbreviations are used in this manuscript:

8-OHdG	8-hydroxy-2′-deoxyguanosine
d-ROMs	Reactive oxygen metabolites
DNA	Deoxyribonucleic acid
HN	Histologic nodal classification
IQR	Interquartile range
KIT	KIT protein expression pattern
c-kit	KIT proto-oncogene
Ki-67	Proliferation marker Ki-67
MCT	Mast cell tumor
MDA	Malondialdehyde
